# Responses to transient receptor potential (TRP) channel agonists in *Chlamydomonas reinhardtii*

**DOI:** 10.1242/bio.053140

**Published:** 2020-07-08

**Authors:** Mamoru Wada, Itaru Kaizuka, Kenjiro Yoshimura

**Affiliations:** Department of Machinery and Control Systems, College of Systems Engineering and Science, Shibaura Institute of Technology, Saitama 337-8570, Japan

**Keywords:** TRP channel, Cilia and flagella, *Chlamydomonas*, Motility, Capsaicin, Gingerol

## Abstract

Pungent substances, such as capsaicin and gingerol, activate the transient receptor potential (TRP)-V1 channel and affect the feeding behaviors of animals. To gain insight into how living organisms have acquired a sense for pungent substances, we explored the response to TRP agonists in a protist, *Chlamydomonas reinhardtii*. When capsaicin or gingerol was applied to wild-type cells, they became immotile, with flagella detaching from the cell body. The degree of deflagellation was nearly halved in a mutant defective in the TRP channel ADF1. Deflagellation in the *adf1* mutant was inhibited further by Ruthenium Red, indicating ADF1 and another TRP channel are involved in the deflagellation response. The response to capsaicin and gingerol was not inhibited by TRPV1-specific blockers such as 4-(3-Chloro-2-pyridinyl)-N-[4-(1,1-dimethylethyl)phenyl]-1-piperazinecarboxamide (BCTC) and capsazepine. When capsaicin or gingerol was applied to wild-type cells in the presence of Ruthenium Red, a large proportion lost motility while flagella remained attached, suggesting that flagella stop contributing to motility, at least in part, through a TRP-channel-independent pathway. These results indicate that pungent compounds such as capsaicin and gingerol induce loss of flagellar motility and flagellar detachment in *C.* *reinhardtii* cells.

## Abstract (precis)

**Summary:** *Chlamydomonas reinhardtii* cells lose motility and excise flagella upon application of pungent TRP channel agonists such as capsaicin and gingerol, likely through TRP channel activity, including ADF1.

## INTRODUCTION

Living organisms have various sensors to detect environmental and bodily conditions. In animals, ion channels called transient receptor potential (TRP) channels respond to chemical, thermal and mechanical stimuli. Mammalian TRP channels are classified into six families, including TRPA, TRPC, TRPM, TRPML, TRPP and TRPV, which are further classified into TRPV1, TRPV2 and so on. The TRPN subfamily is found in fish and invertebrates, but not in mammals. Invertebrates, fungi and protists also have TRP channels, which are difficult to classify using mammalian TRP channel families.

Many TRP channels are activated by pungent substances. For example, capsaicin, a spicy component of pepper, activates TRPV1. TRPV1 is also activated by gingerol, a major pungent component of ginger, and piperine, a bioactive substance in pepper (Vriens et al., 2008). Other examples include TRPA1 activation by allyl isothiocyanate (AITC), a pungent component of wasabi, horseradish and mustard oil, and TRPM8 activation by menthol, a component of peppermint.

Some studies have explored why plants produce such pungent substances. Chili peppers potentially produce capsaicin to protect fruit from consumption by rodents and facilitate the dispersion of seeds by birds (Tewksbury and Nabhan, 2001). When non-pungent chili mutants are given to rodents, they eat and grind chili seeds with their molars, resulting in a low germination rate. Bird TRPV1, on the other hand, lacks sensitivity to capsaicin, although it is activated by heat and acid (Jordt and Julius, 2002). Birds consume pungent chili and disperse seeds to places beneficial to growth without decreasing their germination rate. Another explanation is that capsaicin prevents damage by insects and molds, as its presence in chili fruit protects it from being punctured by Hemiptera and infected by fungal pathogens (Tewksbury et al., 2008).

It is not known how animals have developed a sense for pungent substances. TRPV1 in vertebrates, including human, rat and rabbit, is sensitive to capsaicin at concentrations from 0.1 to 10 µM. Although invertebrates lack TRP channels that can be clearly categorized into TRPV1 (Venkatachalam and Montell, 2007), various responses to capsaicin have been reported. For example, thermal avoidance in *Caenorhabditis* *elegans* is strengthened by applying 100 µM capsaicin (Wittenburg and Baumeister, 1999), while *Drosophila* show behavioral preference to capsaicin (Al-Anzi et al., 2006). Temperature preferences change upon applying 100 µM capsaicin to American cockroach and mealworm larvae (Olszewska, 2010; Olszewska and Tęgowska, 2011; Maliszewska et al., 2018). Capsaicin also inhibits the attachment of mussels through byssus, suggesting that capsaicin could potentially be used as an antifouling biocide (Angarano et al., 2007). Although it has been reported that schistosome motility is affected by capsaicin via the activity of a TRPA1-like channel (Bais et al., 2015), the molecular identity of a capsaicin receptor is largely unknown in invertebrates.

The responses to capsaicin in protists are still poorly understood. To the best of our knowledge, there is only one report describing bioluminescence stimulation by capsaicin in the dinoflagellate *Lingulodinium polyedra* (Lindström et al., 2017). Uncovering protist responses to pungent substances and identifying their receptors would promote our understanding of TRP channel evolution and its role in sensing pungent substances.

*Chlamydomonas reinhardtii* has at least eight TRP channel genes, some of which have been characterized. TRP11 is involved in the avoidance reaction, a mechanoresponse to collision with an obstacle (Fujiu et al., 2011). ADF1 is required for acid-induced deflagellation (Hilton et al., 2016), TRP1 responds to temperature as shown by electrophysiology (Arias-Darraz et al., 2015) and PKD2 is associated with mating (Huang et al., 2007). In terms of the effect of pungent substances, *C. reinhardtii* growth is not affected by capsaicin up to 250 µg l^−1^ (Oliveira et al., 2017).

In this study, we examined the acute responses to pungent substances in *C. reinhardtii*. We found that *C. reinhardtii* flagella detach from the cell body on application of capsaicin or gingerol, and that the ADF1 channel is involved in the deflagellation response.

## RESULTS

### Wild-type *C. reinhardtii* cells respond to TRP channel agonists by deflagellation

**Fig. 1.**
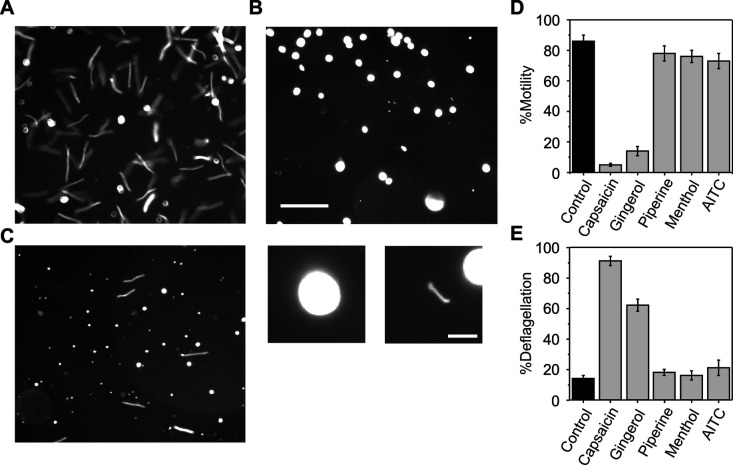
**Responses to TRP channel agonists in wild-type *C. reinhardtii* cells.** (A) Cells in the presence of no TRP channel agonist (A), 200 µM capsaicin (B) and 200 µM gingerol (C). Scale bar: 100 µM. Shutter was kept open for 0.5 s. Enlarged images for a deflagellated cell and a detached flagellum are shown below panel B (Scale bar: 10 µM). (D,E) The proportion of motile cells (%motility) and deflagellated cells (%deflagellation) is shown in D and E, respectively. Mean and standard deviation (s.d.) (*n*=5).

When wild-type *C. reinhardtii* cells were suspended in the experimental solution and observed under a microscope, nearly all of the cells were motile and swam in a linear path (Fig. 1A). When 200 µM capsaicin was applied, most cells became immotile (Fig. 1B). Observation at higher magnification revealed that flagella were detached from the cell body, indicating that the loss of motility was due to deflagellation. When 200 µM gingerol was applied, approximately 90% of cells became immotile, whereas some cells retained motility (Fig. 1C).

The proportion of motile cells and deflagellated cells were counted after the application of various TRP channel agonists. Applying 200 µM capsaicin decreased the proportion of motile cells (%motility) to less than 10% and increased the proportion of deflagellated cells (%deflagellation) to 90% (Fig. 1D,E). Treatment with 200 µM gingerol also reduced %motility to approximately 10% of cells and induced deflagellation in approximately 60% of cells, suggesting that gingerol has an effect similar to capsaicin but is less effective at the same concentration. Piperine had virtually no effect on motility and deflagellation, despite being a human TRPV1 agonist like capsaicin and gingerol. Loss of motility or deflagellation also did not occur with menthol, a TRPM8 agonist, or AITC, a TRPA1 agonist. Thus, capsaicin and gingerol resulted in motility loss and deflagellation while piperine, menthol and AITC did not.

### Deflagellation by capsaicin and gingerol is blocked by TRP channel antagonists

**Fig. 2.**
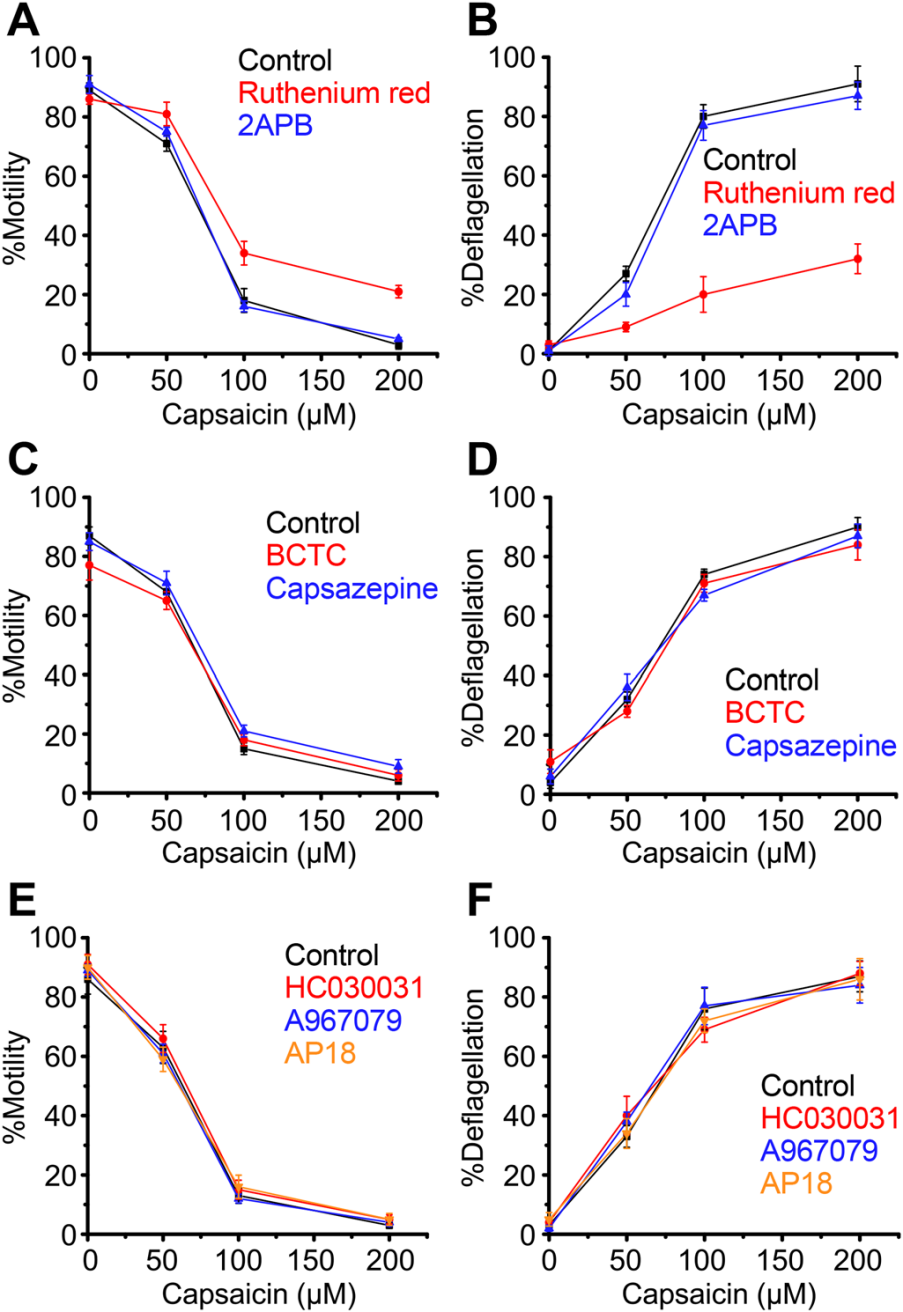
**Loss of motility and flagella upon application of capsaicin to wild-type cells.** Response in the absence of blocker is shown in black. (A,B) Responses in the presence of Ruthenium Red (red) and 2APB (blue). (C,D) Responses in the presence of BCTC (red) and capsazepine (blue). (E,F) Responses in the presence of HC030031 (red), A967079 (blue) and AP18 (orange). Mean and s.d. (*n*=5).

**Fig. 3.**
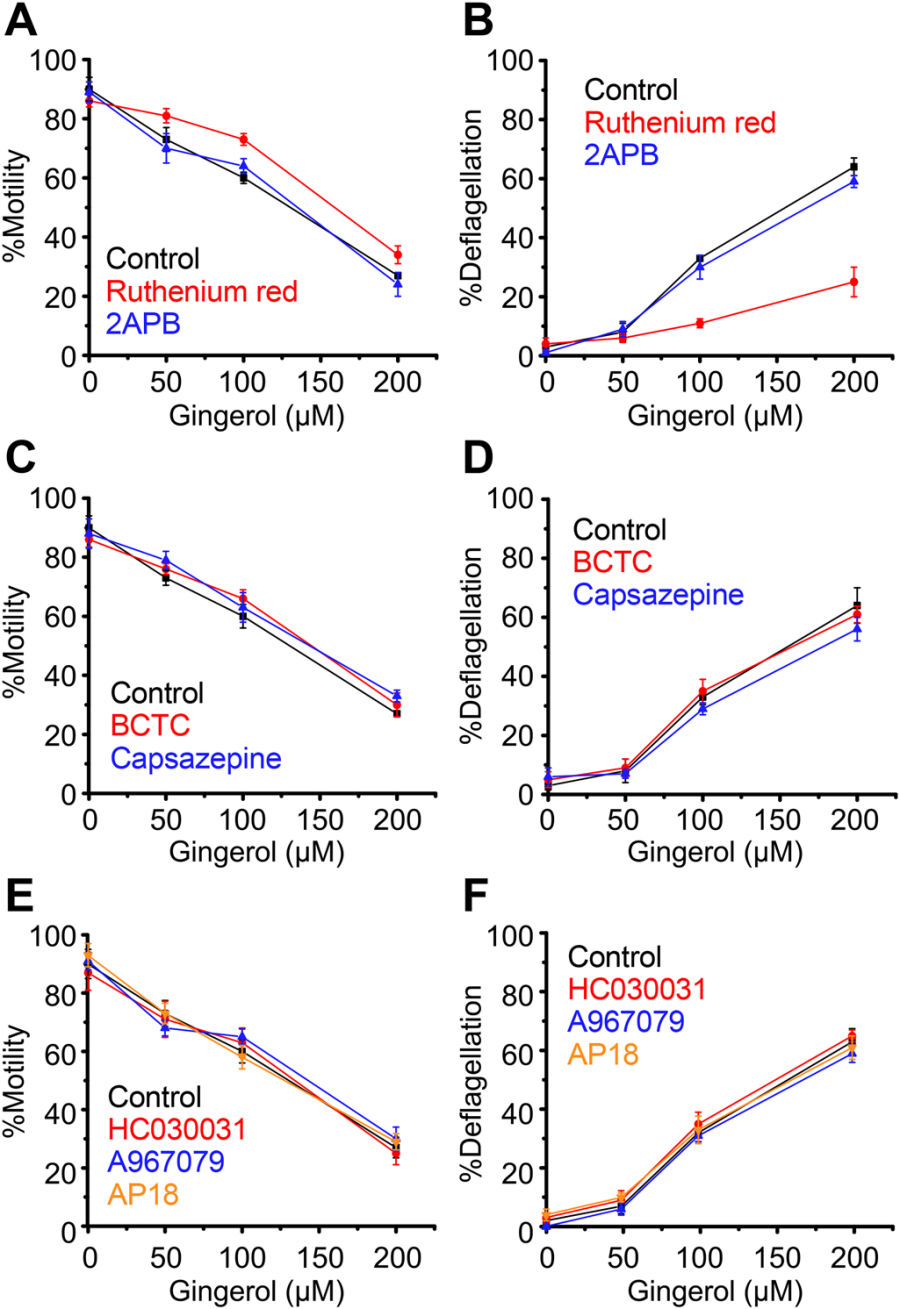
**Loss of motility and flagella upon application of gingerol to wild-type cells.** Response in the absence of blocker is shown in black. (A,B) Responses in the presence of Ruthenium Red (red) and 2APB (blue). (C,D) Responses in the presence of BCTC (red) and capsazepine (blue). (E,F) Responses in the presence of HC030031 (red), A967079 (blue) and AP18 (orange). Mean and s.d. (*n*=5).

When capsaicin was applied at various concentrations, less than half of cells responded at 50 µM and more than half at 100 µM in terms of motility loss and deflagellation, indicating an EC_50_ for capsaicin is between 50–100 µM (Fig. 2A). We next tested whether general TRP channel inhibitors could block capsaicin- and gingerol-induced deflagellation. When capsaicin was applied in the presence of Ruthenium Red, %motility improved by approximately 20% at 200 µM capsaicin (Fig. 2A). %deflagellation at 200 µM capsaicin was markedly reduced from approximately 90% to 30% (Fig. 3B). 2APB did not block capsaicin effects. Since capsaicin and gingerol are activators of animal TRPV1, capsaicin was applied in the presence of inhibitors specific for TRPV1. However, BCTC and capsazepine did not affect motility loss and deflagellation by capsaicin (Fig. 2C,D). Supposing that *C. reinhardtii* TRP channels responding to capsaicin and gingerol do not share TRPV1-inhibitor specificity, TRP-channel blockers specific for other TRP-channel family members were tested. However, capsaicin-induced motility loss and deflagellation was not blocked by HC030031, A967079 or AP18 (Fig. 2E,F).

The effects described above on TRP channels were similarly examined for responsiveness to gingerol. Similar to the capsaicin response, Ruthenium Red improved %motility slightly and greatly reduced %deflagellation after gingerol treatment (Fig. 3A,B). The specific TRP-channel inhibitors BCTC, capsazepine, HC030031, A967079 and AP18 did not affect the response to gingerol (Fig. 3C–F).

Considering that TRP channels are permeable to cations and deflagellation is induced by the elevation of intracellular Ca^2+^ concentration in *C. reinhardtii* (Quarmby and Hartzell, 1994), we tested whether depletion of extracellular Ca^2+^ affects the deflagellation by capsaicin. When CaCl_2_ was omitted and 5 mM EGTA was included in the experimental solution, 200 µM capsaicin treatment resulted in deflagellation in only a small proportion of cells (16.4±12.9%, *n*=5).

### The TRP channel ADF1 is involved in the response to TRP channel agonists

**Fig. 4.**
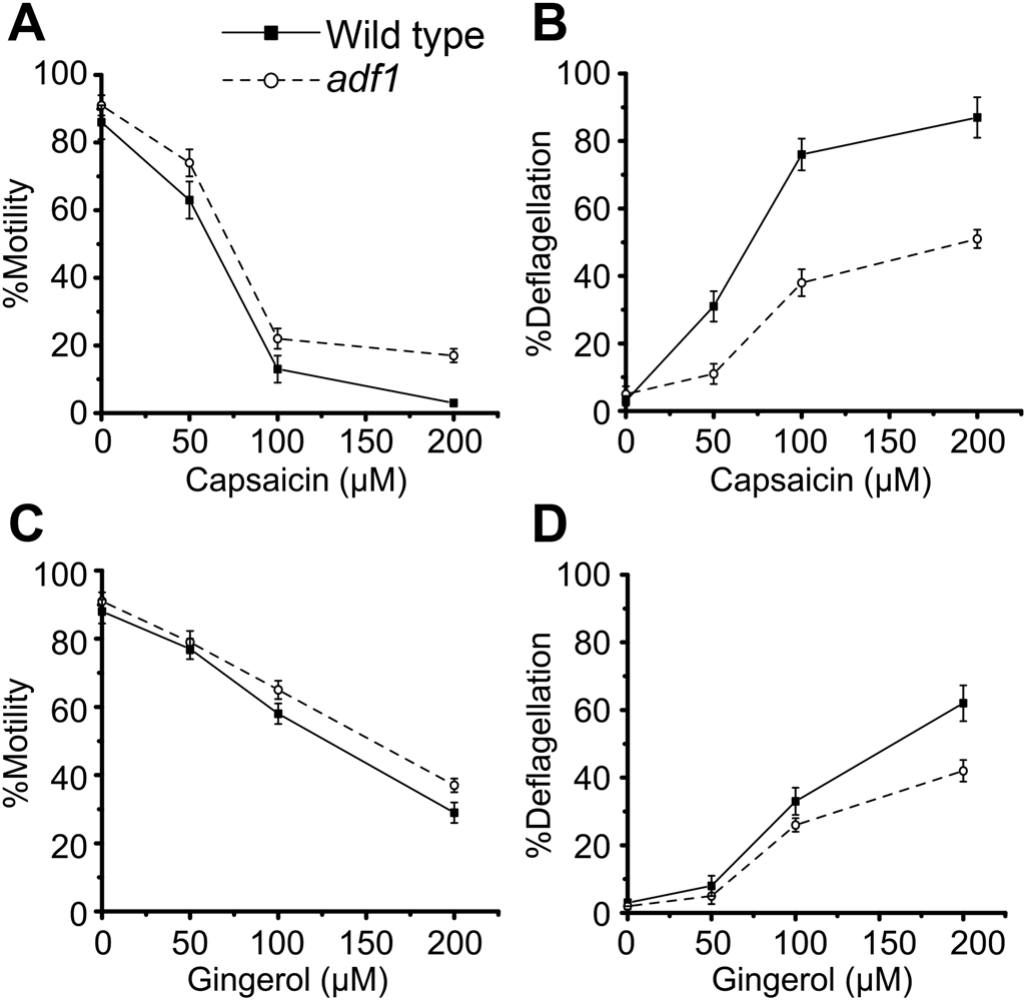
**Effect of capsaicin and gingerol on *adf1* cells.** Responses to capsaicin (A,B) and gingerol (C,D) in wild-type (solid line) and *adf1* (broken line) cells are shown. Mean and s.d. (*n*=5).

When weak acid is applied to *C. reinhardtii* cells, extracellular calcium ions flow into cells and activate flagellar excision machinery located in the transition zone of flagella (Salisbury et al., 1987). *adf1* is a mutant defective in such acid-induced deflagellation because of a mutation in a TRP channel (Hilton et al., 2016). To examine whether capsaicin- or gingerol-induced deflagellation shares the reaction pathway with acid-induced deflagellation, we applied capsaicin and gingerol to *adf1* cells. The proportion of motile cells decreased with increasing capsaicin concentrations in *adf1* cells, although the response was slightly improved compared to wild-type cells (Fig. 4A). However, the proportion of deflagellated cells was almost halved in *adf1* cells compared to wild-type cells (Fig. 4B). Notably, the decrease in deflagellated cells was smaller than that observed with Ruthenium Red treatment (Fig. 2B). This observation indicates that capsaicin-induced deflagellation depends on ADF1, but also TRP channels other than ADF1.

The response to gingerol was less effective in *adf1* cells in terms of motility loss and deflagellation than in wild-type cells (Fig. 4C,D), but the effect of *adf1* mutation was not as evident as observed with capsaicin.

### TRP channels other than ADF1 are also involved in the response to TRP channel agonists

**Fig. 5.**
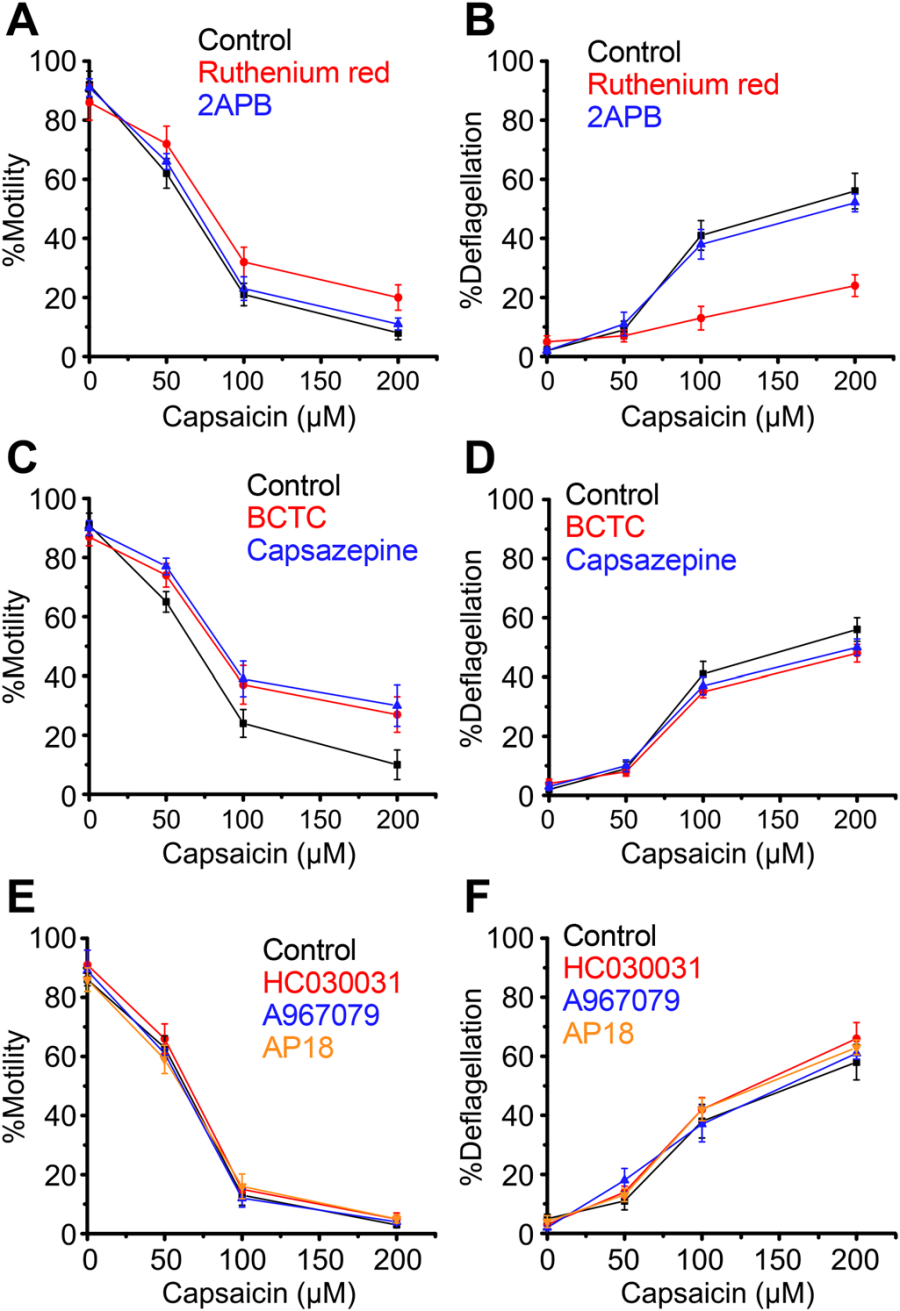
**Loss of motility and flagella upon application of capsaicin to *adf1* cells.** Response in the absence of blocker is shown in black. (A,B) Responses in the presence of Ruthenium Red (red) and 2APB (blue). (C,D) Response in presence of BCTC (red) and capsazepine (blue). (E,F) Response in presence of HC030031 (red), A967079 (blue) and AP18 (orange). Mean and s.d. (*n*=5).

To test the view that TRP channels other than ADF1 are involved in the response to capsaicin and gingerol, we applied capsaicin or gingerol to *adf1* cells in the presence of TRP channel inhibitors. When capsaicin was applied in the presence of Ruthenium Red, although the proportion of immotile cells decreased slightly (Fig. 5A), the proportion of deflagellated cells decreased significantly (Fig. 5B). 2APB did not affect the capsaicin response in *adf1* cells. The proportion of motile cells also increased slightly when BCTC or capsazepine was present, while these inhibitors did not affect deflagellation (Fig. 5C,D). HC030031, A967079 and AP18 did not affect the response to capsaicin in *adf1* cells (Fig. 5E,F).

**Fig. 6.**
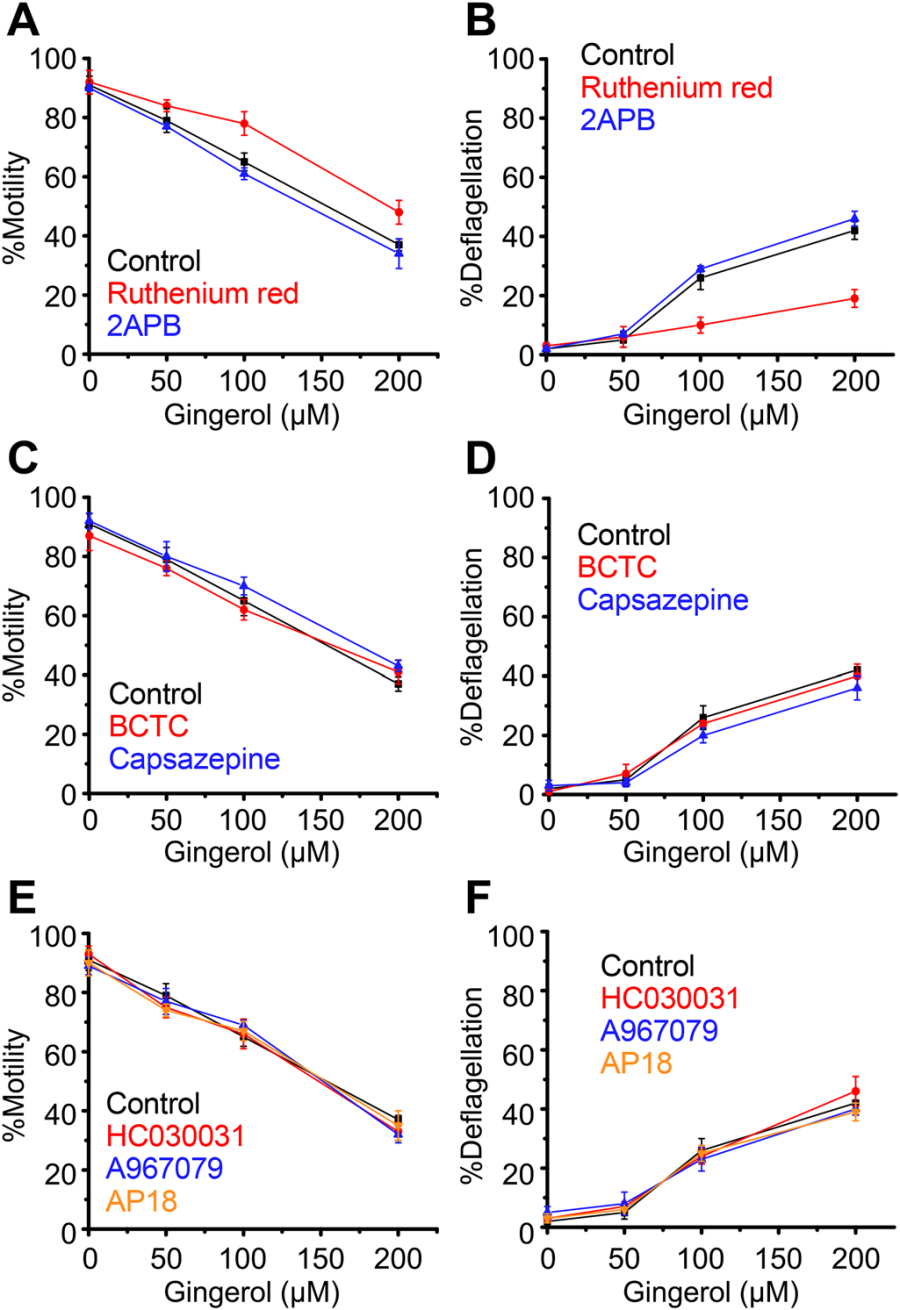
**Loss of motility and flagella upon application of gingerol to *adf1* cells.** Response in the absence of blocker is shown in black. (A,B) Responses in the presence of Ruthenium Red (red) and 2APB (blue). (C,D) Responses in the presence of BCTC (red) and capsazepine (blue). (E,F) Responses in the presence of HC030031 (red), A967079 (blue), and AP18 (orange). Mean and s.d. (*n*=5).

When the effects of TRP channel inhibitors on the gingerol response were examined in *adf1* cells, Ruthenium Red slightly improved motility and greatly reduced deflagellation (Fig. 6A,B). None of the specific TRP channel inhibitors affected the response to gingerol (Fig. 6C–F).

### Deflagellation by low pH shock is blocked by TRP-channel antagonists

**Fig. 7.**
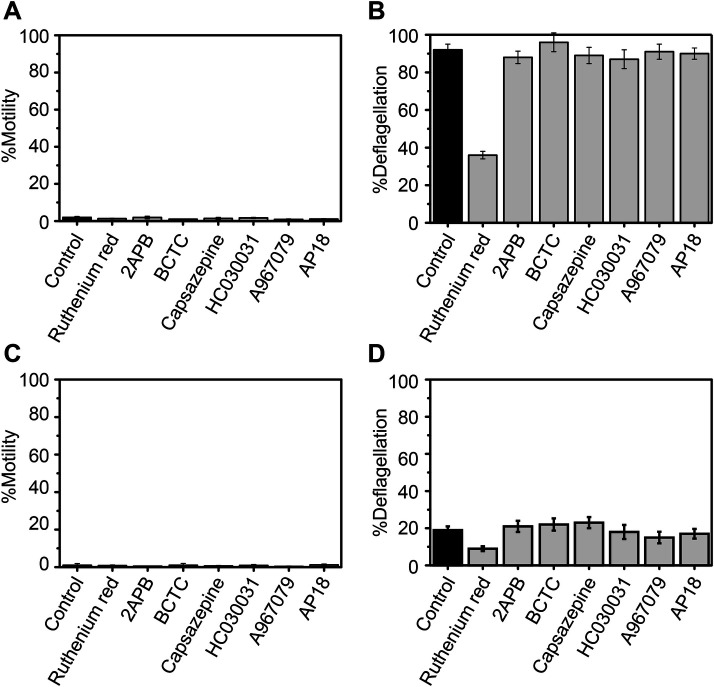
**Effect of TRP channel blockers on the response to low-pH shock in wild-type (A,B) and *adf1* (C,D) cells.** Mean and s.d. (*n*=5).

The effects of TRP-channel-inhibitor treatment and the *adf1* mutation on the response to capsaicin and gingerol implies that ADF1 is sensitive to TRP channel inhibitors. To test this idea, we applied low-pH shock to wild-type and *adf1* cells in the presence of TRP channel inhibitors. When pH shock was applied to wild-type cells, nearly all cells lost motility and approximately 90% of cells lost flagella (Fig. 7A,B). The loss of motility was not affected by any of the inhibitors tested. Deflagellation was not blocked by inhibitors except for Ruthenium Red. When pH shock was applied to *adf1* cells, motility was almost completely lost, but only a small portion (as low as approximately 20%) deflagellated, as reported previously (Finst et al., 1998) (Fig. 7C,D). The motility loss was not recovered by any of the inhibitors. The proportion of deflagellated cells did not change when TRP channel inhibitors were applied except for a small reduction when Ruthenium Red was present.

## DISCUSSION

The present result shows that *C. reinhardtii* flagella detach from the cell body upon application of capsaicin or gingerol. Detachment is probably due to the activation of the flagellar excision machinery present at the base of flagella (Salisbury et al., 1987). Since the excision machinery is activated by the elevation of intracellular calcium ion concentrations (Quarmby and Hartzell, 1994), capsaicin and gingerol likely elevate calcium ion concentrations at the flagellar base. The failure to induce deflagellation by capsaicin in the Ca^2+^-free solution supports the view that an influx of extracellular Ca^2+^ is involved in the elevation of intracellular Ca^2+^ concentration. To the best of our knowledge, there has been no report showing that capsaicin or gingerol induce any responses in flagella or cilia, including deflagellation or deciliation. Tracheal cilia increase beat frequency when capsaicin is applied, but this response is an indirect response that involves neural pathways (Eljamal et al., 1994).

*C. reinhardtii* cells shed flagella upon application of various stimuli such as chemicals (acid, dibucaine, alcohol, mastoparan), temperature change and mechanical shear. The physiological significance of deflagellation in *C. reinhardtii* has been discussed but is still not fully understood. Since most reported chemicals that induce deflagellation are too harsh or artificial to occur in the natural environment, deflagellation may be due to hyperactivation and the overwhelming of a calcium-sensitive break point at the flagellar base, as discussed by Quarmby (2009). Natural substances like capsaicin and gingerol are more likely to be encountered. Although it is not known how high capsaicin or gingerol concentrations can be in natural environments such as soil, other substances may exist that can induce deflagellation at lower concentrations.

The degree of deflagellation upon capsaicin application was reduced significantly in *adf1* cells, indicating that ADF1 is, at least in part, involved in the response to capsaicin. It is plausible that ADF1 is activated directly by capsaicin binding, but also possible that ADF1 is activated indirectly via intracellular messengers, since ADF1 activity is controlled by calmodulin (Wu et al., 2018). As in the case of acid-induced deflagellation, Ca^2+^ influx through ADF1 may induce Ca^2+^ release from intracellular calcium stores that result in deflagellation (Quarmby, 2009).

If capsaicin binds to ADF1, it is reasonable to expect the presence of a capsaicin binding site on the ADF1 molecule. Capsaicin has been shown to bind to a cavity deep in the transmembrane domain of TRPV1 (Cao et al., 2013). Predicting the capsaicin binding site of ADF1 based on TRPV1 structure is not possible due to low homology between ADF1 and TRPV1. The lack of effect by TRPV1-specific blockers also supports the idea that the ADF1 structure is not homologous to TRPV1. Nevertheless, determining a capsaicin binding site in ADF1 may provide a molecular basis for the evolution of capsaicin sensitivity. For instance, a structural determination of *C. reinhardtii* TRP1 revealed that its structure is homologous to TRPC channels but adopts a unique twofold symmetrical rose-shape architecture with a unique lipid binding site (McGoldrick et al., 2019).

ADF1 does not seem to be the only TRP channel that responds to capsaicin and gingerol, since Ruthenium Red decreased the proportion of deflagellated cells in *adf1* (Figs 5B and 6B). Thus, it is likely that another TRP channel besides ADF1 responds to capsaicin and gingerol and is sensitive to Ruthenium Red. Candidates include four TRP channels present in *C. reinhardtii* flagella and other TRP channels that remain to be identified (Fujiu et al., 2011).

Capsaicin induced deflagellation at a concentration of 100 µM or higher. This concentration is similar to concentrations at which capsaicin takes effect in nematodes, bivalves and insects (Wittenburg and Baumeister, 1999; Olszewska, 2010; Olszewska and Tęgowska, 2011; Angarano et al., 2007). However, the concentration needed to activate mammalian TRPV1 is as low as around 1 µM. For example, the EC_50_ for rat TRPV1 is 0.7 µM (Caterina et al., 1997). This discrepancy may indicate that TRP channels have gained higher sensitivity to capsaicin during evolution. In this sense, it may also justify determining the structure of the capsaicin binding site in ADF1.

When capsaicin or gingerol was applied to wild-type cells, the proportion of motile cells decreased in accordance with the proportion of deflagellated cells, indicating the motility loss could be attributed mainly to deflagellation. However, the inhibitory effect of Ruthenium Red over capsaicin was greater in terms of deflagellation than motility. For example, %motility was as low as approximately 20%, while approximately 70% of cells maintained flagella when 200 µM capsaicin was applied to wild-type or *adf1* cells in the presence of Ruthenium Red (Figs 2A,B and 5A,B). The presence of immotile cells with attached flagella implies that immobilization by capsaicin occurred, at least in part, through a mechanism that is insensitive to Ruthenium Red. It is possible that TRP channels are not involved in this flagellar quiescence since the capsaicin-sensitive TRPV1-independent pathway has been reported in mammals (Ziglioli et al., 2009; Gonzales et al., 2014).

This study has shown that capsaicin and gingerol induce the halt and excision of flagella in *C. reinhardtii*. Deflagellation is likely mediated by ADF1 and an unidentified TRP channel. The loss of motility is probably, at least in part, led by a TRP-channel-independent pathway. These responses may be unique in several species of protists, but it is possible that animal cilia and flagella undergo such marked responses. This study reflects the diversity of responses to TRP channel agonists and suggests that *C. reinhardtii* might provide a clue to understanding the evolutionary origin of sensitivity to pungent substances.

## MATERIALS AND METHODS

### Cells

The wild-type *C.* *reinhardtii* (a progeny from the mating of two wild-type strains, CC124 [mt-] and CC125 [mt+], devoid of the *agg1* mutation, Ueki et al., 2016) and *adf1* mutant (CC2919, obtained from the *Chlamydomonas* Resource Center) were used. *adf1* is defective in acid-induced deflagellation (Finst et al., 1998). Cells were grown in Tris-acetate-phosphate medium (Gorman and Levine, 1964) under 12 h light/ 12 h dark conditions at 25°C. Cells were washed twice with an experimental solution containing 5 mM KCl, 0.3 mM CaCl_2_, 0.2 mM EGTA-KOH and 5 mM HEPES-KOH (pH 7.4) by centrifugation (2320 g, 5 min). Ca^2+^-free solution contained 5 mM KCl, 5 mM EGTA-KOH and 5 mM HEPES-KOH (pH 7.4).

### TRP channel agonists and antagonists

The TRP channel agonists used in this study were capsaicin, gingerol, piperine, AITC and menthol. TRP channel inhibitors included 4-(3-Chloro-2-pyridinyl)-N-[4-(1,1-dimethylethyl)phenyl]-1- piperazinecarboxamide (BCTC) (20 µM), capsazepine (20 µM), HC030031 (300 µM), A967079 (30 µM), AP18 (100 µM), Ruthenium Red (10 µM) and 2APB (30 µM). All chemicals were purchased from Fujifilm Wako Pure Chemical (Osaka, Japan) and dissolved with DMSO for the preparation of stock solutions.

For acid-induced deflagellation, cell suspensions were mixed 1:1 with a deflagellation solution containing 40 mM CH_3_COONa and 1 mM CaCl_2_, pH 4.5 (Finst et al., 1998).

### Microscopic observation

Cell suspensions and experimental solutions containing TRP channel agonists or antagonists were mixed at a ratio of 1:1. When both TRP channel agonists and antagonists were applied, cells were first mixed with antagonist and then with agonist. The response was observed with a microscope (BX51, Olympus, Tokyo, Japan) fitted with dark field optics 2 min after mixing. Red light (>600 nm) was used for illumination to avoid a photoresponse. The image was recorded with a digital camera (ORCA-Flash 4.0 V2, Hamamatsu Photonics, Hamamatsu, Japan) using image acquisition software (HC image, Hamamatsu Photonics). Exposure time was set to 0.5 s for counting the number of motile and immotile cells. 102–136 cells were counted in each experiment.
